# Tracking the rate of initiation and retention on isoniazid preventive therapy in a high human immunodeficiency virus and tuberculosis burden setting of Lesotho

**DOI:** 10.4102/sajid.v34i1.10

**Published:** 2019-11-25

**Authors:** Eltony Mugomeri, Dedré Olivier, Wilhelmiena M.J. van den Heever

**Affiliations:** 1 Medical Laboratory Sciences, Africa University, Mutare, Zimbabwe; 2 Department of Health Sciences, Central University of Technology, Bloemfontein, South Africa

**Keywords:** Cox’s proportional hazards function, isoniazid preventive therapy, tuberculosis, uptake of health interventions, Lesotho

## Abstract

**Background:**

Tuberculosis (TB) remains a public health problem, particularly in people living with human immunodeficiency virus (PLHIV). Yet, efforts to reduce TB incidence using isoniazid preventive therapy (IPT) have been curtailed by poor uptake of this intervention. This study reviewed the rate of IPT initiation in the sub-Saharan country of Lesotho, which has one of the highest TB incidences in the world.

**Methods:**

Time to IPT initiation in randomly sampled medical records of PLHIV was analysed using Cox’s proportional hazards regression. Differences in the periods of enrolment into Human immunodeficiency virus (HIV) care were controlled for by considering the year IPT was launched (2011) as the base year and stratifying the medical records into the 2004–2010 cohort (before the launch of IPT) and the 2011–2016 cohort (after the launch).

**Results:**

Out of 2955 patients included in the final analysis, 68.8% had received IPT by the study exit time. However, the overall rate of IPT initiation was 20.6 per 100 person-years, with 135 (6.6%) treatment interruptions. Compared to the 2004–2010 cohort, the 2011–2016 had a significantly (*p <* 0.05) higher rate of initiation (15.8 vs. 27.0 per 100 person-years, respectively). Age group, district category and duration of antiretroviral therapy emerged as the most significant predictors of IPT initiation, while district category and gender significantly predicted IPT therapy interruption.

**Conclusion:**

These findings indicate a high uptake of IPT with a slow rate of implementation. Significant factors associated with disparities in the initiation and interruption of IPT therapy in this study are important for policy review.

## Introduction

Human immunodeficiency virus (HIV) and tuberculosis (TB) have become a global syndemic responsible for nearly 25% of all HIV-associated deaths.^1^ The World Health Organization (WHO)^1^ notes that the incidence of TB in 2017 was about 133 cases per 100 000 global population and of the 1.3 million people who died from TB in 2017 alone, 300 000 were HIV-positive. Despite the considerable effectiveness of isoniazid preventive therapy (IPT), recommended by the WHO^2^ for the prevention of TB in people living with HIV (PLHIV), the rates of initiation and retention on IPT have generally been suboptimal,^3,4,5,6^ thus necessitating more population studies of this intervention.

The reasons for the slow implementation vary by country, with the most cited being poor health care delivery systems, underestimation of potential public health impact of IPT by HIV/TB programme managers and a lack of adequate means to exclude a pre-existing TB infection prior to treatment initiation, among others.^7,8,9,10^ Some countries were not convinced of the benefits of IPT and deferred the implementation of IPT altogether for many years. For example, the Ivory Coast had not implemented IPT by 2014.^11^

The uptake of health interventions, particularly in developing countries, is often slow.^12^ Scholars believe that this can be overcome by closing the ‘know-do gap’, that is, the gap between what is known and what gets implemented.^13,14,15^ Further, Pablos-Mendez and Shademani^15^ note that slow IPT uptake in developing countries may be a symptom of a range of problems in the health systems of these countries, including a lack of evaluation studies. Therefore, the evaluation of IPT uptake is a crucial step to improving the implementation of IPT.

Lesotho is an independent country completely surrounded by South Africa, with about 2.2 million people and an estimated gross domestic product (GDP) per capita of $1000.00, which puts the country into the low-income countries tier.^16^ With a 23.5% adult HIV prevalence rate, the country has the second highest prevalence rate of HIV worldwide^17^ and with 665 TB incidences per 100 000 population,^1^ the country is one of the top three nations with the highest rates of TB worldwide.

The Government of Lesotho (GoL) implemented the 6-month IPT guidelines of the WHO in 2011.^18^ However, ever since its launch, information on the rate of initiation of IPT and the associated factors has remained obscure.^18^ Of note, the country has not provided data for the IPT indicator to the WHO repository from the time the programme was launched.^1^ So, this study assessed the rate of initiation and retention of PLHIV on IPT and the associated factors in the high HIV/TB burden setting of Lesotho with the aim of identifying the barriers to the implementation of IPT in the country.

## Methods

### Study setting

Lesotho is divided into 10 administrative districts, five of which are densely populated and are considered scale-up districts for HIV/TB programmes. The scale-up districts occupy the lowlands of the country, while the other five (non-scale-up districts) occupy the mountainous sparsely populated highlands.^19^

**FIGURE 1:**
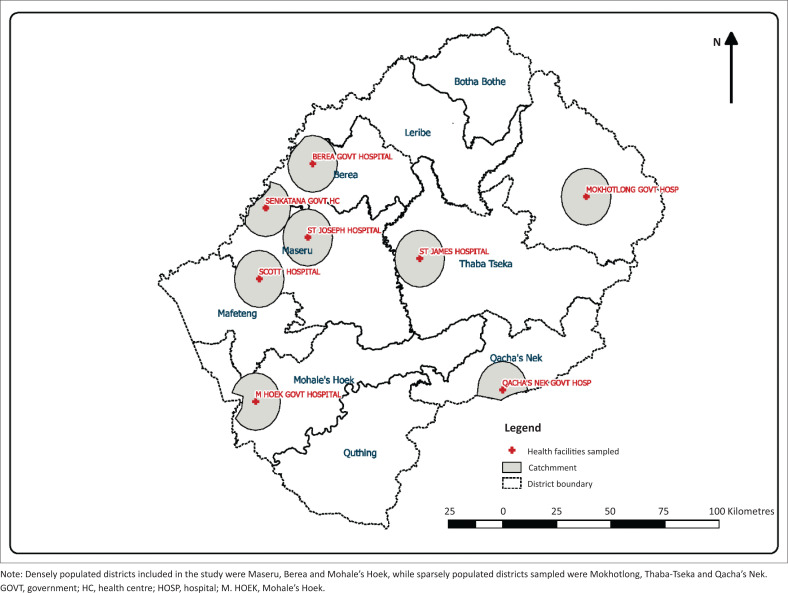
Data collection sites.

Study participants were sampled from three district hospitals in three sparsely populated (non-scale-up) districts and five district hospitals from three densely inhabited (scale-up) districts of the country (see Figure 1).

### Study design

This longitudinal retrospective cohort study analysed, based on Cox’s proportional hazard regression, the probability of IPT initiation in PLHIV in Lesotho, considering time to IPT as the dependent variable. Further, the study assumed that the rate of IPT initiation was fairly constant and that the rate generally fitted an exponential curve that could be modelled by Cox’s proportional hazards function.^4^

### Study population

The target population was PLHIV enrolled into HIV care between 2004 and 2016 in the eight health institutions, all of which were district hospitals with at least 2000 patients on antiretroviral therapy (ART). Antiretroviral therapy records of HIV-positive children, adolescents and adults, including geriatric patients and pregnant women, were selected using stratified systematic random sampling across eight health facilities, ensuring proportional representation of all patient categories including gender, age and period of enrolment.

### Sample size calculation

The minimum sample size was calculated following standard guidelines for estimating incidence with a specified relative precision.^20^ The minimum number of patients’ records, assuming a relative precision of 10% at 5% significance level,^20^ was 385 at each hospital or 3080 in the eight hospitals. However, an additional 40% were added to cater for incomplete records, implying that at least 4620 patient files were required.

### Patient sampling and data collection

File selection was based on stratified systematic random sampling using a sampling frame that was prepared from the ART attendance registers by drawing a list of all the patients enrolled into HIV care since 2004. The total sample of files per hospital was obtained by dividing the total number of patients enrolled in HIV care by the proportional target sample size for the hospital.

**FIGURE 2:**
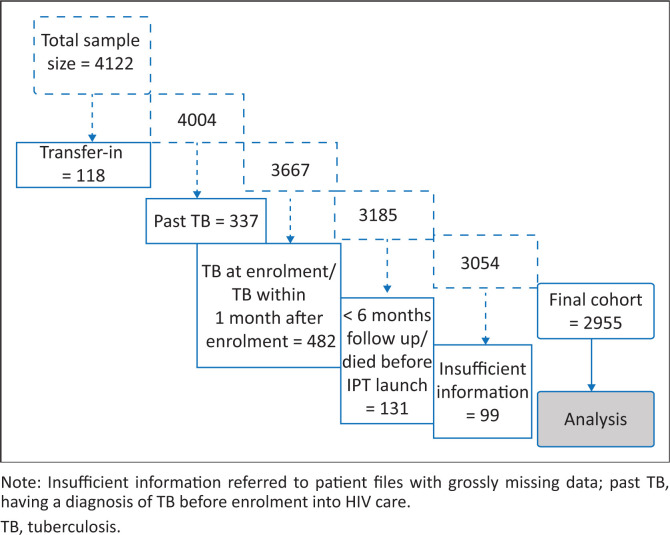
Exclusion criteria for the patients.

Demographic and clinical data were captured between January and October 2016 from the paper-based ART and IPT registers into a Microsoft Access database (Microsoft, Richmond, United States) designed by the researchers. Overall, 4122 patient files were collected. Of the 4122 patients, 1167 were excluded according to the exclusion criteria presented in Figure 2.

### Patient data and outcome measures

Time-variant variables such as age were calculated according to the time at enrolment into HIV care. Patients late for their scheduled appointments by more than a month and those who did not return for ART services were classified as loss to follow-up.

Time to IPT (in months) was calculated by subtracting the date of enrolment into HIV care from the date IPT was prescribed. Patient treatment outcomes were assessed by median cluster of differentiation 4 (CD4) and viral load values. The duration before ART commencement (in years), known as the pre-ART period, was calculated by subtracting the date of HIV diagnosis from that of ART commencement. Furthermore, the proportion of patients who had interrupted IPT therapy was also calculated and the factors associated with interrupting therapy were assessed using chi-square test and logistic regression analysis.

### Data preparation

Data for IPT initiation in the database were verified and exported to Stata version 13.1 (StataCorp, TX, United States) for further cleaning and analysis. Data were formatted for survival analysis as discrete-time survival data with interval date as the time variable. The occurrence of the IPT event defined the ‘failure’ outcome with the time scale in years. To obtain comparable follow-up times, patients enrolled before IPT was available were declared to have entered the risk set in 2011, the year IPT was launched in the country. For purposes of calculating Cox’s proportional hazards ratios, entry times into the risk set for all the patients were delayed by 1 month to calibrate for inconsistencies at first entry into the risk set. The date of enrolment into HIV care and the exit date marked the left and right censoring times, respectively.

### Analysing patient characteristics associated with isoniazid preventive therapy initiation

The proportional ‘hazard’^21^ in this study was the probability that an individual with certain characteristics would receive IPT in a specified time. To cater for observation gaps, follow-up times were subdivided into 6-month discrete intervals^22^ and Breslow’s correction was used to correct for this treatment of data.^21^

Patient characteristics associated with IPT initiation were summarised by cross-tabulation. These characteristics were further analysed using univariate Kaplan–Meier survival functions to determine their suitability in the analysis model. Further, Wilcoxon’s log-rank test and Cox regression analysis were used to determine equality across strata for categorical variables and continuous variables, respectively. Predictors with *p* < 0.2 were included in the IPT initiation model.

The year 2011, when IPT was launched, was set as the base year for follow-up and medical records were stratified into the 2004–2010 cohort (before the launch of IPT) and the 2011–2016 cohort (after the launch of IPT). Ten predictors (see Table 1) were selected into the model and excluded using the stepwise method.

**TABLE 1:** Associations between predictors and isoniazid preventive therapy initiation stratified by period of enrolment into human immunodeficiency virus care.

Variable	Overall			Enrolment period 2004–2010							Enrolment period 2011–2016						
	IPT cases		IPT cases/100 person-years	Total (*N*)	IPT cases Row		Person-years	IPT cases/100 person-years†	Chi-square	p	Total (*N*)	IPT cases Row		Person-years	IPT cases/100 person-years	Chi-squared	p
	%	*N*			%	*n*						%	*n*				
**Gender**	**-**	**-**		**-**		**-**	**-**	**-**	**1.5**	**0.226**	**-**	**-**	**-**	**-**	**-**	**1.8**	**0.176**
Female	72	1394	21.1	798	77	613	3740	16.4	-	-	1144	68	781	2856	27.3	-	-
Male	63	639	19.6	412	68	279	1901	14.7	-	-	601	60	360	1367	26.3	-	-
**Age**	**-**	**-**	**-**		**-**	**-**	**-**	**-**	**3.1**	**0.373**	**-**	**-**	**-**	**-**	**-**	**4.3**	**0.23**
Children	40	21	10.7	31	42	13	135	9.6	-	-	21	38	8	61	13.2	-	-
Adolescents	60	33	28.2	12	42	5	43	11.7	-	-	43	65	28	74	37.6	-	-
Adult	69	1876	20.7	1116	75	839	5217	16.1	-	-	1587	65	1037	3849	26.9	-	-
Elderly	71	103	21.2	51	69	35	246	14.2	-	-	94	72	68	239	28.4	-	-
**District category**	**-**	**-**	**-**	**-**	**-**	**-**	**-**	**-**	**0.9**	**0.336**	**-**	**-**		**-**	**-**	**2.9**	**0.091**
Dense	72	1379	20.5	922	75	691	4266	16.2	-	-	991	69	688	2452	28.1	-	-
Sparse	63	654	20.8	288	70	201	1375	14.6	-	-	754	60	453	1772	25.6	-	-
**Adherence**	**-**	**-**	**-**	**-**	**-**	**-**	**-**	**-**	**0.2**	**0.642**	**-**	**-**	**-**	**-**	**-**	**1.1**	**0.29**
Good	68	1581	21.7	843	75	620	3864	16.0	-	-	1493	68	961	3420	28.1	-	-
Poor	73	452	17.5	367	74	272	1777	15.3	-	-	252	71	180	802	22.4	-	-
**Treatment failure**	**-**	**-**	**-**	**-**	**-**	**-**	**-**	**-**	**2.1**	**0.144**	**-**	**-**	**-**	**-**	**-**	**0.2**	**0.649**
No	69	1993	19.1	1176	73	860	5471	18.9	-	-	1733	65	1133	4184	27.1	-	-
Yes	87	40	20.6	34	94	32	169	15.7	-	-	12	67	8	40	20.2	-	-
**BL CD4**	**-**	**-**	**-**	**-**	**-**	**-**	**-**	**-**	**2.4**	**0.515**	**-**	**-**	**-**	**-**	**-**	**2.7**	**0.313**
1–100	65	413	18.9	321	71	229	1449	15.8	-	-	318	59	186	757	24.6	-	-
101–350	72	1232	19.9	757	76	567	3582	15.8	-	-	957	70	666	2602	25.6	-	-
351–500	60	214	26.2	81	76	62	379	16.3	-	-	276	59	162	477	34.0	-	-
501–1572	66	161	26.0	51	67	34	231	14.6	-	-	194	65	127	387	32.8	-	-
**BL WHO stage**	**-**	**-**	**-**	**-**	**-**	**-**	**-**	**-**	**4.2**	**0.240**	**-**	**-**	**-**	**-**	**-**	**12.4**	**0.006**
I	70	733	24.4	246	80	196	1192	16.4	-	-	803	67	537	1813	29.6	-	-
II	69	842	18.7	609	71	430	2848	15.1	-	-	610	67	412	1664	24.7	-	-
III	73	379	19.6	295	76	224	1355	16.5	-	-	221	70	155	577	26.8	-	-
IV	46	79	19.1	60	70	42	245	17.1	-	-	111	33	37	169	21.9	-	-
**Duration of pre-ART**	**-**	**-**	**-**	**-**	**-**	**-**	**-**	**-**	**1.9**	**0.602**	**-**	**-**	**-**	**-**	**-**	**4.5**	**0.213**
< 1	70	1491	20.7	892	74	663	4151	15.9	-	-	1235	67	828	3050	27.2	-	-
1–2	74	198	21.1	121	76	92	561	16.4	-	-	148	72	106	377	28.1	-	-
3–5	67	229	19.2	135	76	102	654	15.6	-	-	209	56.4	127	538	23.6	-	-
> 5	53	115	21.6	62	56	35	273	12.8	-	-	153	46.6	80	258	31.0	-	-
**Duration of ART (years)**	**-**	**-**	**-**	**-**	**-**	**-**	**-**	**-**	**24.0**	**0.000**	**-**	**-**	**-**	**-**	**-**	**9.4**	**0.024**
0–2	45	329	59.4	59	2	1	40	2.4	-	-	678	48.4	328	514	63.8	-	-
3–4	67	479	23.7	63	32	20	191	10.4	-	-	654	70.2	459	1830	25.1	-	-
5–6	81	570	17.6	313	76	239	1499	15.9	-	-	388	85.3	331	1745	19.0	-	-
> 6	82	655	16.2	775	81	632	3909	16.1	-	-	25	92.0	23	134	17.1	-	-
**Total**	**69**	**2033**	**20.6**	**1210**	**74**	**892**	**5641**	**15.8**	**-**	**-**	**1745**	**65**	**1141**	**4223**	**27.0**	**300**	**0.000†**

Note: CD4 counts are in cells/mm^3^.

ART, antiretroviral therapy; BL, baseline; IPT, isoniazid preventive therapy; *N*, number of patients; TB, tuberculosis; tnd, target not detected; WHO, World Health Organization. CD4 counts are in cells/mm^3^.

† , The observation time in 100 person-years is only for tallying purposes because IPT was launched in 2011.

The model was tested for predictor interaction and two variables namely, duration of ART and the district category had significant interactions (*p <* 0.005). The models with and without the interaction variables were compared using the likelihood ratio test (Lrtest) and the difference was found to be significant (Chi [3] = 10.0; *p* = 0.006), implying that the bigger model with the interaction terms was superior to the one without.

The unstratified model was checked for proportionality using the Schoenfeld and scaled Schoenfeld residuals (Phtest) test. One categorical variable, duration of ART significantly (*p* = 0.032), violated the proportionality assumption, but was, however, retained in the model and the anomaly corrected through stratification with period of enrolment.

### Ethical considerations

The study was approved by the Ethics Committee of the Ministry of Health of Lesotho. Permission to conduct the study was also granted by the hospital authorities. Fictional identifier codes were assigned to the patients in the database and all patient data were treated with confidentiality.

## Results

### Associations between incident isoniazid preventive therapy initiation and predictor variables

In total, 2955 patients were included in the final analysis (see Appendix 1, Table 1-A1, for extended patient characteristics cross-tabulated with IPT initiation outcome). Table 1 presents the overall incident IPT cases and the cases stratified by period of enrolment. By proportion, 68.8% of the patients received IPT during follow-up, while 31.2% had not received the drug at the exit time for the study. Overall, 2033 incident IPT cases in 9728 person-years of observation occurred out of the 2955 patients in the study. The overall rate of IPT initiation was 20.6 per 100 person-years. The effective follow-up time since IPT was launched in 2011 ranged from 0.5 to 5.8 years [mean = 3.5; median = 4.1; interquartile range [IQR]: 1.4–5.6]. With the data stratified by period of enrolment, the median time to IPT for the patients enrolled before 2011 was higher than that of the patients enrolled on ART after 2011 (4.8 vs. 2.5 years, respectively). Compared to patients enrolled before 2011, patients enrolled after 2011 had a significantly (*p* = 0.000) higher initiation rate (15.8 vs. 27.0 per 100 person-years, respectively).

Four variables, namely gender, antiretroviral treatment failure, baseline WHO clinical stage and duration on ART, had considerable association (*p <* 0.300) with IPT initiation in the patients enrolled on ART before 2011. However, for the patients enrolled on ART after 2011, more variables, namely, gender, age, district category, adherence, baseline WHO clinical stage, duration on pre-ART and duration on ART had substantial influence (*p <* 0.300) on IPT initiation.

**FIGURE 3:**
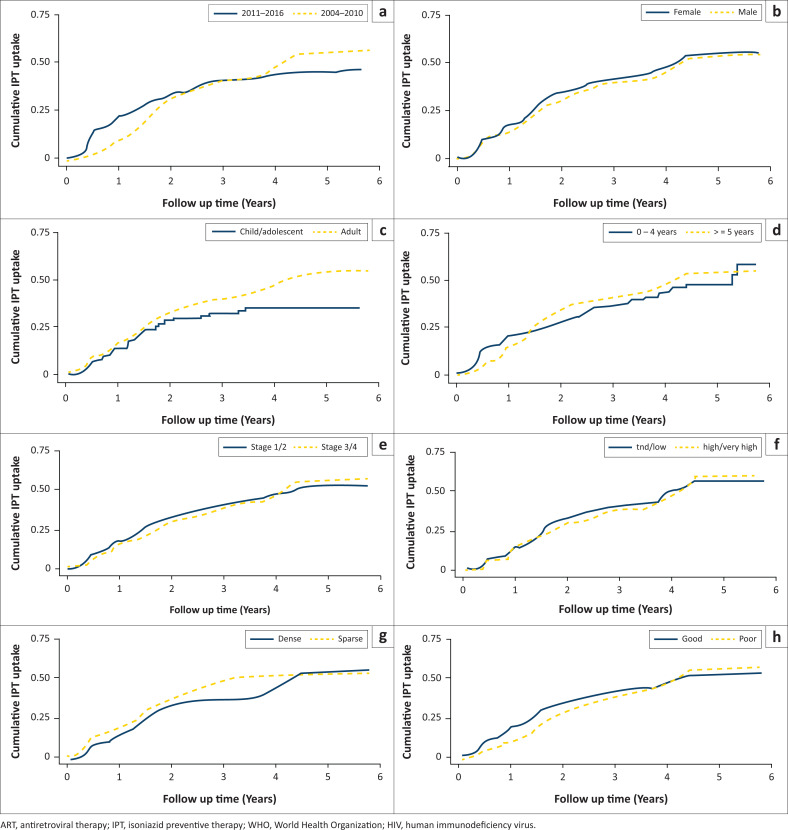
(a) Kaplan–Meier function of time to isoniazid preventive therapy initiation by predictor variables. Age group (c) and duration of antiretroviral therapy (d) were important determinants of isoniazid preventive therapy initiation, while gender (b) was not. Baseline World Health Organization clinical stage (e), median viral load (f), district category (g) and adherence (h) were all important predictors of isoniazid preventive therapy initiation.

Figures 3(a) and 3(b) depict the predictive effect of selected variables in Table 1 on IPT initiation based on the Kaplan–Meier failure function of time to IPT initiation per predictor variable. Period of enrolment and district category were disproportionate, which justified the need for stratified analysis with period of enrolment as the strata variable to correct this anomaly.

Significant (*p <* 0.2) categorical predictor variables that had distinct plots were namely: (1) gender, (2) marital status, (3) district, (4) geographic location, (5) period of enrolment into HIV care, (6) baseline WHO clinical stage, (7) adherence to ART, (8) patient status at exit time of the study and (9) history of past TB infection on first visit. For continuous predictors, significant (*p <* 0.2) predictors (in Table 1) that also had distinct plots (Figures 3a and 3b) were namely: (1) age, (2) baseline CD4 count, (3) median CD4 count, (4) median viral load and (5) duration of pre-ART.

### Associations between patient characteristics and interrupting isoniazid preventive therapy

Table 2 presents the associations between patient characteristics and interrupting IPT therapy. Gender and the district density category were significantly (*p <* 0.050) associated with interrupting IPT by chi-square test, while baseline CD4 count was only marginally significant (*t* = 7.6; *p* = 0.056). Multiple logistic regression analysis revealed that although baseline CD4 count category 351 cells/mm^3^ – 500 cells/mm^3^ (odds ratio [OR]: 2.2; 95% confidence interval [CI]: 1.2–4.0; *p* = 0.013) and baseline WHO clinical stage IV (OR: 2.5; 95% CI: 1.2–5.1; *p* = 0.015) were also significant, sparsely populated district category (adjusted OR: 1.6; 95% CI: 1.1–2.3; *p* = 0.000) and gender (adjusted OR: 2.1; 95% CI: 1.5–3.0; *p* = 0.006) were the most influential in determining interruption of IPT therapy.

**TABLE 2:** Association between patient characteristics and isoniazid preventive interrupted therapy.

Patient characteristic	Total (*n*)	Interrupted IPT		Chi-square	*p*	Univariate			Multivariate		
		Row %	*n*			Unadjusted OR	(95% CI)	*p*	Adjusted OR	(95% CI)	*p*
**Age**	**-**	**-**	**-**	**4.1**	**0.765**	**Excluded**	**-**	**-**	**Excluded**	**-**	**-**
0–9	21	0	0	-	-	-	-	-	-	-	-
9–18	22	9.1	2	-	-	-	-	-	-	-	-
18–25	179	5.6	10	-	-	-	-	-	-	-	-
25–35	691	6.2	43	-	-	-	-	-	-	-	-
35–45	598	7.4	44	-	-	-	-	-	-	-	-
45–55	338	7.7	26	-	-	-	-	-	-	-	-
55–65	151	6	9	-	-	-	-	-	-	-	-
65–84	33	3	1	-	-	-	-	-	-	-	-
**Gender**	**-**	**-**	**-**	**7.8**	**0.005**	**base**	**1**	**-**	**base**	**1**	**-**
Female	1394	5.6	78	-	-	-	-	-	-	-	-
Male	639	8.9	57	-	-	1.7	1.2–2.4	0.006	1.7	1.5–3.0	0.006
**Geographic location**	**-**	**-**	**-**	**18.5**	**0.000**	**base**	**1**	**-**	**base**	**1**	**-**
Dense	1379	5	69	-	-	-	-	-	-	-	-
Sparse	654	10.1	66	-	-	2.1	1.5–3.0	0.000	1.6	1.1–2.3	0.000
**Period enrolled into HIV care**	**-**	**-**	**-**	**0.2**	**0.689**	**base**	**1**	**0.689**	**Excluded**	**-**	**-**
2011–2016	1141	6.8	78	-	-	-	-	-	-	-	-
2004–2010	892	6.4	57	-	-	1.1	0.8–1.5	-	-	-	-
**Baseline WHO clinical stage**	**-**	**-**	**-**	**7.6**	**0.056**	**base**	**-**	**base**	**-**	**-**	**-**
1	733	6.4	47	-	-	-	-	-	-	-	-
2	842	6.7	56	-	-	1.0	0.7–1.6	0.848	1.3	0.9–2.1	0.174
3	379	5.5	21	-	-	0.9	0.5–1.5	0.566	1.1	0.6–2.0	0.688
4	79	13.9	11	-	-	2.4	1.2–4.8	0.016	2.5	1.2–5.1	0.015
**BL CD4 count**	**-**	**-**	**-**	**13.1**	**0.004**	**base**	**1**	**base**	**1**	**-**	**-**
1–100	415	5.8	24	-	-	-	-	-	-	-	-
101–350	1233	5.7	70	-	-	0.9	0.6–1.6	0.936	1.1	0.6–1.7	0.828
351–500	224	11.6	26	-	-	2.1	1.2–3.8	0.010	2.2	1.2–4.0	0.013
501–1512	161	9.3	15	-	-	1.7	0.9–3.3	0.133	2.0	2.0–3.9	0.060

Note: CD4 counts are in cells/mm^3^.

IPT, isoniazid preventive therapy; *n*, number of patients; ART, antiretroviral therapy; BL, baseline; WHO, World Health Organization; HIV, human immunodeficiency virus; OR, odds ratio; CI, confidence interval.

### The rate of initiation of isoniazid preventive therapy

Out of 10 variables considered for inclusion in the model, three predictors, district category, age group and duration on ART emerged as significant (*p <* 0.050) predictors (Table 3). Two predictors, duration on ART and district category, had significant (*p* = 0.000) interactions (shown as duration on ART # District category in Table 3).

**TABLE 3:** Cox proportional hazards model for initiation of isoniazid preventive therapy by people living with human immunodeficiency virus in Lesotho.

Predictor	Total (*N*)	Outcome: Initiation rate per 100 PY	Unstratified model: 2004–2016						Model stratified by period of enrolment			
			Unadjusted HR		*p*	Adjusted HR		*p*	2004–2010		2011–2016	
			HR	95% CI		HR	95% CI		Adjusted HR	95% CI	Adjusted HR	95% CI
**Enrolment period**												
2011–2016	1745	27.0	1	(base)		1	(base)		-	-	-	-
2004–2010	1210	15.8	0.60	0.54–0.68	0.000	0.63	0.55–0.72	0.000	-	-	-	-
**Duration of pre-ART**												
< 1	2127	20.7	1	(base)	-	1	(base)	-	-	-	-	-
1–2	269	21.1	1.02	0.86–1.20	0.851	1.01	0.85–1.20	0.909	-	-	-	-
3–5	344	19.2	1.06	0.91–1.23	0.491	0.99	0.85–1.16	0.905	-	-	-	-
> 5	215	21.6	0.96	0.76–1.20	0.706	0.84	0.66–1.06	0.137	-	-	-	-
**Baseline WHO stage**												
I	1049	24.4	1	(base)	-	1	-	-	-	-	-	-
II	1219	18.7	0.81	0.72–0.91	0.000	0.87	0.77–0.98	0.023	-	-	-	-
III	516	19.6	0.83	0.72–0.96	0.011	0.92	0.79–1.06	0.255	-	-	-	-
IV	171	19.1	0.88	0.68–1.13	0.333	0.92	0.72–1.19	0.538	-	-	-	-
**Adherence**												
Good	2219	21.7	1	(base)		1	-	-	-	-	-	-
Poor	615	17.5	0.96	0.85–1.07	0.432	0.96	0.85–1.07	0.462	-	-	-	-
**District category**												
Sparse	1042	16.9	1	(base)	-	1	-	-	1	(base)	1	(base)
Dense	1913	15.4	0.77	0.69–0.86	0.000	0.58	0.42–0.78	0.000	1.03	0.46–2.30	0.59	0.38–0.94
**Duration on ART†**												
0–4	1454	16.0	1	(base)	-	1	(base)	-	1	(base)	-	-
³5	1501	15.8	0.76	0.67–0.86	0.000	1.40	1.16–1.70	0.001	3.34	2.06–5.43	1.33	1.00–1.76
**Geographic location # Duration of ART**	**-**	**-**	**-**	**-**	**-**	**1**	**(base)**	**-**	**1**	**(base)**	**-**	**-**
Sparse	1042	16.9	-	-	-	0.81	0.83–0.95	0.000	0.86	0.77–0.9	0.95	0.85–1.06
Dense	1913	15.4	-	-	-	0.86	0.79–0.89	0.000	0.86	0.81–0.9	1.01	0.89–1.14
**Gender†**												
Female	1942	16.1	1	(base)	-	1	(base)	-	-	-	-	-
Male	1013	15.4	0.97	0.87–1.08	0.568	0.97	0.88–1.07	0.579	-	-	-	-
**Age†**												
Child/adolescent	107	9.7	1	(base)		1	(base)	-	1	(base)	1	(base)
Adult	2848	16.1	1.63	1.13–2.33	0.008	1.71	1.20–2.47	0.003	1.64	1.02–2.6	1.78	1.00–3.15
**Baseline CD4 count†**												
1–100	639	15.9	1	(base)	-	1	(base)	-	-	-	-	-
101–350	1714	15.9	1.10	0.98–1.25	0.116	1.08	0.95–1.22	0.231	-	-	-	-
351–500	357	17.3	1.25	1.03–1.52	0.024	1.11	0.91–1.35	0.316	-	-	-	-
501–1572	245	14.6	1.07	0.85–1.36	0.553	1.02	0.81–1.30	0.841	-	-	-	-
**Treatment failure†**												
No	2909	18.2	1	(base)	-	1	(base)	-	-	-	-	-
Yes	46	15.9	0.74	0.53–1.04	0.083	0.79	0.56–1.11	0.176	-	-	-	-

WHO, World Health Organization; ART, antiretroviral therapy; *N*, number of patients; PLHIV, people living with HIV; # denotes interaction of term; CI, confidence interval; HR, Hazard Ratio.

† , Predictors insignificant when controlled for baseline WHO clinical Stage, duration of ART, district category and adherence to ART.

In the final model stratified by period of enrolment into HIV care (Table 3), considering the patients enrolled on ART before 2011, the following findings were noted: (1) adults had a 64% higher probability of receiving IPT (Hazard Ratio [HR] = 1.64; 95% CI: 1.02–2.61) in relation to children and adolescents, (2) the likelihood of receiving IPT did not statistically differ between patients in the densely populated districts (HR = 1.03; 95% CI: 0.46–2.30) and those in the sparsely populated regions and (3) longer durations on ART were associated with higher chances of IPT uptake. For example, patients on ART for 5 or more years were three times more likely to receive IPT compared to patients on ART for less than 5 years (HR = 3.34; 95% CI: 2.06–5.43). However, comparing two subjects on ART for 5 or more years in the densely populated districts and considering the interaction terms, having 5 or more years of ART was 20% more likely to receive IPT compared to patients on ART for less than 5 years.

Considering the patients enrolled in the 2011–2016 period, notable trends were as follows: (1) adults had 78% higher probability of receiving IPT (HR = 1.78; 95% CI: 1.00–3.15) in relation to children and adolescents; (2) patients in the densely populated districts had 59% lower likelihood of receiving IPT (HR = 0.59; 95% CI: 0.38–0.94) compared to the sparsely populated districts and (3) longer durations on ART were still associated with higher chances of IPT initiation. For instance, patients on ART for 5 or more years were 33% more likely to receive IPT compared to patients on ART for less than 5 years (HR = 1.33; 95% CI: 1.00–1.76). However, comparing two subjects on ART for 5 or more years in the densely populated districts and taking into account the interaction terms, having 5 or more years of ART was 34% more likely to receive IPT compared to patients on ART for less than 5 years.

## Discussion

The study found a high IPT uptake (68.8%) with generally a slow IPT initiation rate of 20.6 per 100 person*-*years. This indicates that despite the majority of PLHIV getting initiated on IPT, the rate of IPT implementation remains slow. More importantly, this study also indicates the need for reporting the rate of initiation for IPT in addition to national coverage statistics as is the current practice globally. Currently, data on rate IPT initiation are scarce, with only Brazil reporting such data – the rate of initiation in that country was 20.0 per 100 person*-*years in 2014.^4^ Widespread tracking of the rate of initiation for IPT in developing countries would certainly need more bioinformatic tools and skills that are often lacking in these countries.

Despite the lack of comparable data on the rate of IPT initiation from other countries in the southern African region and beyond, IPT coverage in Lesotho is commendable and encouraging. To put IPT coverage for Lesotho into perspective, WHO^1^ reports that national coverage for IPT in 15 of the 30 high HIV/TB burden countries ranged from 1% in Swaziland to 53% in South Africa. Other countries in sub-Saharan Africa with IPT coverage higher than 30% include Ethiopia (45%), Nigeria (39%), Sierra Leone (22%), Zambia (18%), Namibia (15%) and Angola (13%). Notably, IPT coverage in many sub-Saharan countries, such as Botswana, Malawi, Ghana and Uganda, was unknown in 2017.^1^ Therefore, considering the high TB burden in Lesotho, there is need to maintain the high uptake rate for IPT while improving the slow rate of initiation to ensure that newly ART-initiated patients are given IPT as early as possible.^23^

Given that IPT is projected to be more effective in high TB burden countries when optimally implemented,^24^ this study further highlights that Lesotho is losing the opportunity to control TB through IPT. With an incidence rate of TB exceeding 600 cases/100 000 population,^24^ Lesotho needs to intensify the scaling up of IPT.

The median time to IPT for the 2004–2010 cohort was almost twice as high as that of the 2011–2016 cohort, implying that the patients enrolled on ART after IPT was launched have a higher initiation rate in this setting. This disparity therefore needs to be addressed.

The fact that children and adolescents had a lower chance of IPT initiation compared to adults in both the 2004–2010 and 2011–2016 cohorts emphasises the need for scale-up efforts for these patient groups. Tadesse et al.^7^ and Triasih et al.^25^ in Ethiopia and Indonesia, respectively, note that children in resource-limited high TB burden settings have a low initiation rate of IPT despite that a 6-month course of IPT reduces the risk of childhood TB by the same margin of about 60% as adults. Lesotho therefore needs to scale-up TB contact screening as recommended by WHO.^23^ Deficiencies for the identification of children in need of IPT have also been reported in Brazil, Benin and Indonesia,^25,26,27^ and these include health care worker and health facility-related factors, including social support and access.

Isoniazid preventive therapy initiation in the densely populated districts was much lower in the 2011–2016 cohort. Access to HIV/TB services including IPT in the densely populated districts has generally been slow because of a number of reasons including resource limitations for scale-up efforts in these areas that have an estimated 72% of the country’s population.^19^ The lower initiation rates in the densely populated districts indicate that IPT scale-up efforts, currently supported by President’s Emergency Plan for AIDS Relief (PEPFAR) Lesotho, 19 need to be intensified in these districts.

Interrupting of IPT therapy in this study was associated with staying in the sparsely populated districts, being male, having baseline CD4 count of 351 cells/mm^3^ – 500 cells/mm^3^ and baseline WHO clinical stage IV. Patients in the sparsely populated districts, particularly those who are bedridden and in WHO clinical stage IV, may be interrupting therapy because of long distances from the hospitals and the mountainous terrain associated with these geographic locations. Scaling up health care delivery in these areas is therefore needed to reduce therapy interruption. In South Africa, inefficient health service delivery, ineffective communication with health care workers and the financial burden of transport to clinic were the most significant factors for interrupting IPT therapy.^28^ Higher rates of treatment interruption among men emphasise the need for intensified patient education in this group. In Botswana, men also had more interrupted IPT therapy than women.^29^

A qualitative assessment of the factors contributing to low IPT initiation is required in this setting. Of note, barriers to IPT implementation in other settings include health care worker inexperience, unawareness of the benefits of IPT, as well as poor understanding of IPT guidelines and TB screening tools.^30^ Therefore, there is a need to assess the effect of health care worker factors on implementation of IPT in Lesotho.

Of note, in some African countries such as the Ivory Coast, the national guidelines had not started using IPT by 2014, citing that IPT could lead to resistant TB bacilli in patients with undiagnosed TB.^11^ This highlights the magnitude of the challenge of scaling up this intervention in developing countries.

This study is not without limitations, one of which is the lack of data on patient views. As Ostermann et al.^12^ note, interventions must factor in the preferences of the intended target populations to improve initiation and adherence to the intervention. Therefore, investigations on patient preferences and concerns about IPT are needed in Lesotho. However, the main strength of this study is the reporting of IPT initiation in person-years which allows comparisons with other settings outside Lesotho. This study also demonstrates that routine data in ART programmes of developing countries can be useful for inferential analysis.

## Conclusion

This study investigated the rate of IPT initiation from the time the IPT programme was launched in 2011 in a high TB burden setting of Lesotho. Despite the high overall IPT uptake, the rate of IPT initiation was slow. The slow initiation of IPT is disconcerting and the factors associated with poor rate of initiation in this study need to be addressed. The high rate of IPT treatment interruption in the sparsely inhabited districts is evidence to the need to improve the monitoring of this programme. Clearly, the implementation of this health intervention in a high TB burden setting of Lesotho needs further scale-up.

## Appendix 1

**TABLE 1-A1:** Associations between patient characteristics and isoniazid preventive therapy initiation in people living with human immunodeficiency virus in Lesotho.

Variable	Total		Received IPT while in HIV care					
			No		Yes		Chi-square	*p*
	Column %	*n*	Row %	*n*	Row %	*n*		
**Gender**							23.5	0.000
Female	65.7	1942	28.2	548	71.8	1394	-	-
Male	34.3	1013	36.9	374	63.1	639	-	-
**Age (years)**							30.8	0.000
0–9	1.8	52	59.6	31	40.4	21	-	-
9–18	1.3	38	42.1	16	57.9	22	-	-
18–25	9.1	269	33.5	90	66.5	179	-	-
25–35	34.8	1027	32.7	336	67.3	691	-	-
35–45	27.9	824	27.4	226	72.6	598	-	-
45–55	16.1	477	29.1	139	70.9	338	-	-
55–65	7.6	224	32.6	73	67.4	151	-	-
65–84	1.5	44	25	11	75	33	-	-
**Marital status**							24.7	0.000
Married	59.3	1752	32.2	564	67.8	1188	-	-
Divorced	0.4	11	27.3	3	72.7	8	-	-
Separated	6.5	193	26.9	52	73.1	141	-	-
Widowed	15.5	458	25.5	117	74.5	341	-	-
Cohabiting	0.2	6	33.3	2	66.7	4	-	-
Single	14.9	440	37.7	166	62.3	274	-	-
(-)	3.2	95	18.9	18	81.1	77	-	-
**District**							235.9	0.000
Berea	18.3	540	12.4	67	87.6	473	-	-
Maseru	38.7	1145	29.8	341	70.2	804	-	-
Mohales Hoek	7.7	228	55.3	126	44.7	102	-	-
Mokhotlong	11.4	336	20.2	68	79.8	268	-	-
Qachas Nek	10.2	302	45.7	138	54.3	164	-	-
Thaba-Tseka	13.7	404	45	182	55	222	-	-
**District category**							-	0.000
Dense	64.7	1913	27.9	534	72.1	1379	27.3	
Sparse	35.3	1042	37.2	388	62.8	654	-	-
**Period of enrolment into HIV care**							23.1	0.000
2011–2016	59.1	1745	34.6	604	65.4	1141	-	-
2004–2010	40.9	1210	26.3	318	73.7	892	-	-
**Entry point**							50	0.000
Adolescent clinic	0.4	12	0	0	100	12	-	-
HTC	4	118	10.2	12	89.8	106	-	-
MCH	9.1	268	29.5	79	70.5	189	-	-
OPD	77.7	2295	31.2	717	68.8	1578	-	-
Self-refer	7.9	232	44.4	103	55.6	129	-	-
Under-five clinic	0.2	6	50	3	50	3	-	-
Hospital ward	0.8	24	33.3	8	66.7	16	-	-
**Baseline regimen**							22.2	0.005
TDF+3TC+EFV	58.6	1732	30.2	523	69.8	1209	-	-
AZT+3TC+EFV	23.8	704	35.1	247	64.9	457	-	-
AZT+3TC+NVP	5.9	173	27.2	47	72.8	126	-	-
D4T+3TC+NVP	4.1	120	24.2	29	75.8	91	-	-
D4T+3TC+EFV	2.8	84	27.4	23	72.6	61	-	-
TDF+3TC+NVP	2.2	65	26.2	17	73.8	48	-	-
ABC+3TC+NVP	1.1	32	37.5	12	62.5	20	-	-
ABC+3TC+EFV	1	29	55.2	16	44.8	13	-	-
ABC+3TC+kaletra	0.5	16	50	8	50	8	-	-
**Final outcome**							185.9	0.000
Loss	9	265	48.7		51.3	136	-	-
Dead	4.1	121	78.5	129	21.5	26	-	-
Transfer out	2.6	76	35.5	95	64.5	49	-	-
ART continuing	84.4	2493	26.9	27	73.1	1822	-	-
**Adherence to ART**							6.5	0.011
Good	79.1	2336	32.3	755	67.7	1581	-	-
Poor	20.9	619	27	167	73	(452	-	-
**Treatment failure**							7.2	0.007
No	98.4	2909	31.5	916	68.5	1993	-	-
Yes	1.6	46	13	6	87	40	-	-
**BL CD4 count**							19.5	0.000
1–100	-	-	35.1	224	64.9	415	-	-
101–350	-	-	28.1	481	71.9	1233	-	-
351–500	-	-	37.3	133	62.7	224	-	-
501–1512	-	-	34.3	84	65.7	161	-	-
**BL WHO clinical stage**							46.5	0.000
I	-	-	30.1	316	69.9	733	-	-
II	-	-	30.9	377	69.1	842	-	-
III	-	-	26.6	137	73.4	379	-	-
IV	-	-	53.8	92	46.2	79	-	-
**Median viral load**							14.7	0.005
Tnd†	9.1	268	24.3	65	75.7	203	-	-
Low	1.3	38	34.2	13	65.8	25	-	-
High	2.1	61	31.1	19	68.9	42	-	-
Very high	2.8	84	17.9	15	82.1	69	-	-
(-)	84.7	2504	32.3	810	67.7	1694	-	-
**Length of pre-ART (years)**							28.8	0.000
< 1	72	2127	29.9	636	70.1	1491	-	-
01–2	9.1	269	26.4	71	73.6	198	-	-
03–5	11.6	344	33.4	115	66.6	229	-	-
> 5	7.3	215	46.5	100	53.5	115	-	-
**Total**	**100**	**2955**	**31.2**	**922**	**68.8**	**2033**	**-**	**-**

(-), missing information; ART, antiretroviral therapy; ABC, abacavir; AZT, zidovudine; 3TC, lamivudine; kaletra, lopinavir or ritonavir; NVP, nevirapine; EFV, efavirenz; TDF, tenofovir; BL, baseline; HTC, HIV testing and counselling; MCH, mother and child health; OPD, outpatient department.

† , Tnd, target not detected; WHO, World Health Organization; CD4 counts are in cells/mm^3^; viral load ranges in copies/mm^3^ are as follows; tnd (0–50); low (50–500); high (500–10 000) and very high (>10 000).
